# The relationship between body mass index and preeclampsia: A systematic review and meta-analysis

**DOI:** 10.18502/ijrm.v20i12.12568

**Published:** 2023-01-09

**Authors:** Yousef Moradi

**Affiliations:** Social Determinant of the Health Research Center, Research Institute for Health Development, Kurdistan University of Medical Sciences, Sanandaj, Iran.

##  Dear Editor

We studied the article written by Morteza Motedayen et al. (1) that was published in the International Journal of Reproductive Biomedicine in July 2019. The objective of this study was to determine the relationship between body mass index and preeclampsia. The results of this meta-analysis showed that there is a significant relationship between BMI and the risk of preeclampsia, so it can be said that BMI may be one of the ways to diagnose preeclampsia. Although these results were very interesting, some methodological issues should be considered:

Authors for assessing risk of bias and quality of included studies used the Newcastle-Ottawa checklist, but the authors have not reported results of this scale in tables and results.

In the method section, the authors used funnel plot and Beggs test for reporting publication bias, but results related to Beggs test, for example, Beta, p-value, and 95% CI were not reported in the manuscript. Also, for assessing of publication bias in the meta-analysis, it is better to use the Egger test for publication bias, because this test use regression for detecting the correlation between variables and its SD (2-4).

Search strategy is not complete, because this step in meta-analysis should be done independently by 2 researchers. In meta-analysis studies search strategy, quality assessment or risk of bias, and data extraction should be done independently by 2 researchers. On the other hand, gray's literature is not done by authors. The database was incomplete, Web of sciences was not mentioned or searched. Therefore, it seems that in this meta-analysis, the search strategy was not sensitive.

One of the most important aspects of meta-analysis is to determine whether heterogeneity exists in the studies, and investigate the source of such heterogeneity. In this meta-analysis, this section is ambiguous (5). Results showed that heterogeneity is higher and authors have not conducted any subgroup analysis for detecting heterogeneity sources.

In the analysis section, the authors should be calculated the weight or standard mean difference in 2 groups of case-control studies and estimated this standard mean difference in meta-analysis (6, 7). For each study, authors should calculate the standardized mean difference (difference in mean outcomes between groups / standard deviation of outcome among participate). After that, authors should combine these effects and report the pooled estimates (8, 9).

## References

[B1] Motedayen M, Rafiei M, Rezaei Tavirani M, Sayehmiri K, Dousti M (2019). The relationship between body mass index and preeclampsia: A systematic review and meta-analysis. Int J Reprod BioMed.

[B2] Macaskill P, Walter SD, Irwig L (2001). A comparison of methods to detect publication bias in meta‐analysis. Stat Med.

[B3] Peters JL, Sutton AJ, Jones DR, Abrams KR, Rushton L (2006). Comparison of two methods to detect publication bias in meta-analysis. JAMA.

[B4] Doucouliagos H, Stanley TD (2009). Publication selection bias in minimum‐wage research? A meta‐regression analysis. British Journal of Industrial Relations.

[B5] Cooper H, Hedges LV (1994). The hand book of research synthesis.

[B6] Bland JM, Kerry SM (1998). Statistica notes. Weighted comparison of means BMJ.

[B7] Karchevsky M, Babb JS, Schweitzer ME (2008). Can diffusion-weighted imaging be used to differentiate benign from pathologic fractures? A meta-analysis. Skeletal Radiol.

[B8] Devine EC, Westlake SK (1995). The effects of psychoeducational care provided to adults with cancer: Meta-analysis of 116 studies. Oncol Nurs Forum.

[B9] Schwarzer G (2007). Meta: An R package for meta-analysis. R News.

